# The moral concerns of biobank donors: the effect of non-welfare interests on willingness to donate

**DOI:** 10.1186/s40504-016-0036-4

**Published:** 2016-03-11

**Authors:** Raymond G. De Vries, Tom Tomlinson, H. Myra Kim, Chris D. Krenz, Kerry A. Ryan, Nicole Lehpamer, Scott Y. H. Kim

**Affiliations:** Center for Bioethics and Social Sciences in Medicine (CBSSM), University of Michigan, 2800 Plymouth Road, North Campus Research Complex (NCRC), B16-419 W, Ann Arbor, MI 48109-2800 USA; Center for Ethics and Humanities in the Life Sciences, Michigan State University, C-223 East Fee Hall, 965 Fee Road Rm C-208, East Lansing, MI 48824 USA; Center for Statistical Consultation and Research, University of Michigan, 3550 CSCAR, Ann Arbor, MI 48109-1070 USA; Department of Sociology, Michigan State University, 509 E. Circle Dr., Rm 316 Berkey, East Lansing, MI 48824-1111 USA; Department of Bioethics, Clinical Center, National Institutes of Health , 10 Center Drive, 1C118, Bethesda, MD 20892-1156 USA; Department of Psychiatry, University of Michigan, 4250 Plymouth Rd., Ann Arbor, 48109-2700 MI USA

**Keywords:** Biobank, Biobank donors, Bioethics, Blanket consent, Moral concerns, Non-welfare interests, Research attitudes questionnaire, Willingness to donate

## Abstract

Donors to biobanks are typically asked to give blanket consent, allowing their donation to be used in any research authorized by the biobank. This type of consent ignores the evidence that some donors have moral, religious, or cultural concerns about the future uses of their donations – concerns we call “non-welfare interests”. The nature of non-welfare interests and their effect on willingness to donate to a biobank is not well understood.

In order to better undersand the influence of non-welfare interests, we surveyed a national sample of the US population (in June 2014) using a probability-based internet panel. Logistic regression models assessed the demographic and attitudinal characteristics associated with participants’ willingness to give consent for unspecified future uses of their donation when presented with 7 research scenarios that raised possible non-welfare interest concerns.

Most people had non-welfare interests that significantly affect their willingness to donate to a biobank using blanket consent. Some non-welfare interests are associated with subgroups but others are not. A positive attitude toward biomedical research in general was associated with increased willingness to donate, while concerns about privacy and being African American were associated with decreased willingness.

Non-welfare interests matter and can diminish willingness to donate to a biobank. Our data suggest that trust in research promotes willingness to donate. Ignoring non-welfare interests could erode this trust. Donors’ non-welfare interests could be accommodated through greater transparency and easier access to information about the uses of donations.

## Introduction

Biobanking is becoming an increasingly important platform for medical, and specifically genetic, research conducted in the United States and around the world. Having access to curated, centralized repositories of biospecimens, along with their associated demographic and clinical data, allows researchers to perform more efficient and less costly studies, shortening the pipeline from concept to clinical care. Biobanked samples have already been used to understand the genetics of multiple sclerosis (The International Multiple Sclerosis Genetics Consortium 2011), to improve treatments for conditions such as prostate cancer (Akamatsu et al. 2012), to identify a causal link between HPV and cervical cancer (Lehtinen et al. 1996; Wallin et al. 1999), and to study the genomics of cancer formation (George et al. 2015). As the value of genetically-based personalized medicine expands, so too will the role of biobanking (Hewitt 2011).

The promise of biobank research must, however, be balanced against the risk of controversy created when people become passive participants in research about which they have no direct knowledge. A well-known example is the research conducted by Arizona State University researchers using blood collected from members of the Havasupai tribe. Although the blood was originally collected for research on diabetes, the consent was designed to cover any “behavioral/medical” research. When the tribe later discovered that their samples were also used to look for genetic drivers of schizophrenia, they were offended and angry (Van Assche et al. 2013). A more recent case occurred in Texas where the Department of State Health Services – like many state departments of health – routinely collects newborn bloodspots. Parents of newborns there were upset when they learned that researchers were using the bloodspots without their consent or knowledge. Their concern was exacerbated by the fact that researchers could connect genetic information from the blood spots to other personal information in the state’s possession. The parents successfully sued the researchers and more than 5 million samples were destroyed (Lewis et al. 2012).

These cases make visible public concerns about research and exemplify the ways our current ethical and legal frameworks lag behind advances in science and technology. Certain types of research that use biobanked samples, cloning and genetic modification among them (Baker 2014; Liang et al. 2015), create worries about “playing God,” violating privacy, and discrimination by employers and insurers (Bates et al. 2005; Kaufman et al. 2009; Lemke et al. 2010; McDonald et al. 2014; Shabani et al. 2014; Trinidad et al. 2010). The lack of adequate protections for donors intensifies these worries, generating public dissatisfaction with, and distrust of, the research community. Diminished trust in the work of science poses a significant threat to its future in terms of funding and willingness to participate in research.

At the center of this challenge is the question of how best to inform biobank donors about the kinds of research that might use their tissue—a challenge that begins with the initial consent. Biobanks store biospecimens from a variety of sources for future, as yet unknown, research. Neither the biobank donors nor the biobank know with any certainty what kinds of research might eventually use their donated samples—a challenge to the traditional notion of “informed” consent. In light of this, many large population biobanks have adopted a model where the donor consents to future unknown uses of the specimen, without any additional specific consent, and with no provision of information about how the donation has been used (see for example, www.mayo.edu/research/documents/biobank-consent-formpdf/DOC-10027511).

At present, various terms are used to describe this type of consent. The term “blanket consent” is sometimes used to mean completely unregulated consent with no oversight mechanisms at all (Knoppers et al. 2013), with “broad consent” referring to consent for unspecified uses with some degree of oversight (Grady et al. 2015). Others use “broad consent” as a generic reference to any type of consent that covers a range of future uses (including blanket consent) (Gefenas et al. 2012; Petrini 2010). We chose to test a model portraying “blanket consent” with “committee oversight” (see description below in Methods) as a way of focusing on the ethical issue of consenting to future unknown uses of biospecimens – the central issue in the conversation about informed consent for biobanking.

Biobanks take care to de-identify the samples in order to protect the interests and welfare of donors. As such, any analysis conducted using the specimen will not directly affect the donor. Under this model, the donor is generally not even aware that research using their specimen is being conducted. For these reasons, many have argued that a blanket consent model is sufficient to protect participants and uphold standards of informed consent (Rothstein 2005; Office for Human Research Protections 2008)—a position that aligns with proposed changes to the Common Rule (Office for Human Research Protections 2015).

However, this ethical framework is problematic because it does not address the moral, religious, or cultural concerns that donors may have about how their donations are used—concerns we refer to as “non-welfare interests” (NWIs) (Tomlinson 2013; Tomlinson et al. 2014). Current research regulations are primarily concerned with protecting people from various kinds of harms; that is, protecting their welfare interests. Our use of the term “non-welfare interest” highlights concerns that are deeply important to research participants but that are ignored in current regulations. The Havasupai, for example, had a non-welfare interest in how their donations were used. If they had not learned about the schizophrenia research, arguably no harm would have come to them. But because they found out, we now know that they cared deeply about how their samples were used. If they had been told about other possible research using their donated blood, they may have withheld their consent. Existing research has shown that, although the public is largely comfortable with authorizing the use of their samples for unspecified future research (Wendler 2006), they also have significant moral concerns about *how* their specimens might be used—even if they are unaware of such uses (Gornick et al. 2014; Wendler 2006).

Using a nationally representative sample of the public, we recently showed that NWIs do indeed influence willingness to donate to a biobank. When presented with potential research scenarios that raised moral concerns – e.g., related to abortion, genetic research, and biological weapons – our respondents’ willingness to donate to a biobank using a blanket consent was associated with NWIs, and sometimes diminished significantly (Tomlinson et al. 2015).

Given the convincing evidence that NWIs do exist and do influence willingness to donate to biobanks, it is critically important that we understand the drivers of these concerns. If we are to ensure that all segments of the population are represented in biobanks we must be able to respond to the concerns that disincline certain people to donate (McDonald et al. 2014). In this paper, we closely examine the demographic and attitudinal characteristics associated with the influence of NWIs on willingness to donate.

## Materials and Methods

### Study population

A nationally representative sample of the US population was surveyed in June 2014 to explore the effects of non-welfare interests on the willingness to donate to a biobank. Participants were recruited from a probability-based internet panel (KnowledgePanel®) managed by GfK Knowledge Networks, a survey research firm. KnowledgePanel consists of about 55,000 adults (ages 18 and older), representing a probability sampling of 97 % of U.S. households. It recruits households by randomly selecting residential addresses using “address-based sampling” (ABS) which provides statistically valid representation of the U.S. population. The firm’s recruitment and statistical weighting methods are described elsewhere in greater detail (GfK Knowledge Networks 2013).

Of the 2,654 eligible panel members contacted, 1,638 completed the survey. After excluding data from 39 respondents who either answered fewer than half of the survey questions or who completed the survey in less than 2 min, the current study examines data from 1,599 respondents (60.2 % response rate). Compared to non-respondents, respondents were somewhat older, were more likely to be white, and had higher levels of education and household income. A description of the socio-demographic characteristics of respondents and non-respondents can be found elsewhere (Tomlinson et al. 2015). All results, including descriptive statistics, were weighted to correct for stratified sampling designs, non-coverage, and non-response.

The study was reviewed by the Institutional Review Boards at the University of Michigan and Michigan State University and deemed exempt from federal regulations.

### Survey measures

Our survey was developed to explore the effects that non-welfare interests might have on the willingness to donate to a biobank, as well as preferences for biobank consent policies (not discussed here). The survey contained a brief introductory description of the function, purpose, and potential societal benefits of a fictional biobank, as well as a description of ‘blanket consent’ as follows:

“…you will let the biobank use your sample in any study done by researchers who use the biobank. The biobank cannot predict what kind of projects will be done in the future. Thus, it cannot know which future projects will use your sample when you donate. It may happen that your sample is never used… Before a researcher can use your biobank sample, a committee must review the study. This committee will make sure the study is well designed, protects your privacy, and will help society.”

Following the introduction, we used a short 6-item true/false questionnaire to test subjects’ comprehension of the introductory biobank description. The average number of correct responses was 5.1.

As a baseline for blanket consent, respondents were asked to rate how strongly they agreed or disagreed (on a 6-point scale, 1 = Strongly Disagree, 6 = Strongly Agree) with this willingness to donate statement: “I would donate tissue samples and medical information to the biobank, so that it can use them for any research study that it allows, without further consent from me.”

After that baseline question, respondents were introduced to a description of non-welfare interests as follows:

“Research using biobanked samples will help others in the future because it may lead to better ways of finding and preventing disease. For example, it may help researchers find treatments for diseases like cancer. However, some types of research that could be done with biobanked samples might worry some donors because the research might conflict with their religious, cultural, or philosophical beliefs.”

They were then asked to rate their willingness to provide blanket consent “even if” researchers might use their samples in each of 7 (randomly ordered) research scenarios presenting moral concerns. The scenarios were based on potential NWI concerns identified by others (People Science & Policy Ltd 2003; Haddow et al. 2007; National Research Council and Institute of Medicine 2005; Pfeffer 2008; Selgelid 2009; Tomlinson 2009) and described research to:

1. Develop more safe and effective abortion methods (Abortion);
2. Develop kidney stem cells. The goal would be to grow human kidneys or other organs in a pig that could then be transplanted into people (Xenotransplant);
3. Develop patents and earn profits for commercial companies. Most new drugs used to treat or prevent disease come from commercial companies (Patents);
4. Develop stem cells that have the donor’s genetic code. Scientists might use those stem cells to create many different kinds of tissues and organs for use in medical research (Stem cells);
5. Create vaccines against new biological weapons. The government might need to develop biological weapons of its own when it does this research (Bioweapons);
6. Understand the evolution of different ethnic groups, and where they come from. What they learn might conflict with some religious or cultural beliefs (Evolution);
7. Discover genes that make some people more violent. This could lead to ways to reduce violent behavior. But if these genes are found to be more common among some racial and ethnic groups, this might increase prejudice (Violence gene).

We also collected a number of demographic and attitudinal variables (see Table 1) including a measure of “residual privacy concern,” i.e., how worried respondents would be that an unauthorized person might see their private information, even after being told a “committee will make sure the study…protects your privacy” (on a 5-point scale, 1 = “Not worried at all”, 5 = “Very Worried”), and their opinion of biomedical research in general (using the RAQ – Research Attitudes Questionnaire) (Rubright et al. 2011).

**Table 1:** Socio-demographics of participants by dichotomized willingness to donate using blanket consent (baseline)^a^ and total sample

	Agree 1083 (68 %)	Disagree 510 (32 %)	Total 1593	*p*-value^b^
Age (years), mean (SD)	48.8 (16.5)	46.5 (15.1)	48.1 (16.1)	.02
Female	569 (68.6 %)	261 (31.4 %)	830 (52.1 %)	.64
Race				< .001
White	888 (70.9 %)	364 (29.1 %)	1252 (78.6 %)	
Black/African American	92 (48.9 %)	96 (51.1 %)	188 (11.8 %)	
Other^c^	103 (67.5 %)	50 (32.5 %)	153 (9.6 %)	
Hispanic	135 (58.8 %)	95 (41.2 %)	230 (14.5 %)	.001
Education				< .001
< High School	104 (56.2 %)	81 (43.8 %)	185 (11.6 %)	
High school	304 (64.5 %)	167 (35.5 %)	472 (29.7 %)	
Some college	305 (68.0 %)	144 (32.0 %)	448 (28.3 %)	
≥ Bachelor’s Degree	370 (75.8 %)	118 (24.2 %)	487 (30.5 %)	
Household income^d^				< .001
< $50,000	408 (62.1 %)	249 (37.9 %)	656 (41.2 %)	
$50,000–$99,999	349 (69.4 %)	154 (30.6 %)	503 (31.6 %)	
> $100,000	326 (75.3 %)	107 (24.7 %)	433 (27.2 %)	
Attend religious service				.03
≥ Once a month	420 (65.7 %)	219 (34.3 %)	639 (40.4 %)	
< Once a month	374 (73.1 %)	138 (26.9 %)	511 (32.3 %)	
Never	286 (66.2 %)	146 (33.8 %)	432 (27.3 %)	
Religion				< .001
Catholic	246 (71.8 %)	97 (28.2 %)	343 (21.7 %)	
Non-Catholic Christian	485 (67.8 %)	230 (32.2 %)	715 (44.9 %)	
Non-Christian Religions	61 (75.5 %)	20 (24.6 %)	81 (5.1 %)	
Unaffiliated	255 (71.4 %)	102 (28.6 %)	357 (22.6 %)	
Do not know/Refused	33 (36.2 %)	57 (63.8 %)	90 (5.7 %)	
Evangelical	260 (66.0 %)	134 (34.0 %)	395 (41.7 %)	.04
Political view				.004
Liberal	327 (74.9 %)	109 (25.1 %)	437 (27.6 %)	
Moderate	394 (65.8 %)	205 (34.2 %)	599 (37.9 %)	
Conservative	354 (64.8 %)	193 (35.2 %)	546 (34.5 %)	
Region				.02
Northeast	185 (64.2 %)	103 (35.8 %)	288 (18.1 %)	
South	391 (66.1 %)	201 (33.9 %)	592 (37.2 %)	
West	250 (67.3 %)	121 (32.7 %)	372 (23.4 %)	
Midwest	256 (75.3 %)	84 (24.7 %)	340 (21.4 %)	
Employment status				.001
Working	643 (70.6 %)	268 (29.4 %)	911 (57.2 %)	
Looking for work/laid off	83 (53.9 %)	71 (46.1 %)	154 (9.7 %)	
Retired	196 (71.8 %)	77 (28.2 %)	273 (17.2 %)	
Not working, disabled	66 (58.1 %)	48 (41.9 %)	114 (7.2 %)	
Not working, other	95 (67.3 %)	46 (32.7 %)	141 (8.9 %)	
Ownership of housing				.008
Owned	779 (70.2 %)	331 (29.8 %)	1109 (69.7 %)	
Rented	276 (64.7 %)	151 (35.4 %)	428 (26.9 %)	
Occupied w/o cash rent	28 (49.7 %)	28 (50.3 %)	56 (3.5 %)	
Household has internet	881 (70.6 %)	368 (29.4 %)	1248 (78.4 %)	< .001
Privacy^e^, mean (SD)	2.6 (1.2)	3.6 (1.2)	2.9 (1.3)	< .001
RAQ^f^, mean (SD)	46.0 (6.9)	38.1 (7.8)	43.5 (8.1)	< .001
Abortion view^g^, mean (SD)	2.6 (1.0)	2.4 (1.0)	2.4 (1.0)	.001

*N* = 1,593; Cell values are weighted counts (%) or weighted means (SD); Other variables collected, but are not included in the table are not associated with participant position on blanket consent: marital status (*p* = 0.21), head of household (*p* = 0.47), household size (*p* = 0.37), metropolitan area (*p* = 0.93), housing type (*p* = 0.48), whether household members include at least one child (*p* = 0.12)

^a^“I would donate tissue samples and medical information to the biobank, so that the biobank can use them for any research study that it allows, without further consent from me. Please indicate your level of agreement with the statement:”

^b^From survey weight adjusted Х-square test for comparisons between those who agreed vs. not to blanket consent for all participant characteristics and survey weight adjusted *t*-test for age and RAQ

^c^American Indian, Alaskan Native, Asian, Native Hawaiian/Pacific Islander, 2+ races

^d^Collapsed from 19 levels

^e^Range is 1 to 5 (higher is more worried)

^f^RAQ is the 11 item Research Attitudes Questionnaire, assessing attitudes toward medical research. Range is 11–66 (higher score means more positive attitudes)

^g^Range is 1 (always legal) to 4 (always illegal)

### Statistical analysis

The primary outcome variable of interest was willingness to donate. For blanket consent and each of the seven scenarios with NWI concerns, we dichotomized the level of agreement with the “willingness to donate” statement – ranging from 1 to 6 – to “willing” (scores of 4, 5 or 6) and “unwilling” (1, 2 or 3). To understand the effect of potential donors’ socio-demographic characteristics and their attitudes on willingness to donate in the different NWI scenarios, a separate logistic regression model of willingness was fit for each of the seven “non-welfare interests” research scenarios. We considered all participant characteristics (summarized in Table 1) as potential predictors of interest. In order to determine the nature of the relationships – linear or non-linear – between predictors and our outcome variable, we first fit all potential predictors that were continuous or ordinal as categorical dummies. If we found a strong linear relationship – i.e., if the parameter estimates for the categorical dummy variables increased incrementally – we included that variable as a continuous variable. For example, privacy was included in the model as a single continuous variable ranging from not worried at all (1) to very worried (5). Similarly, political affiliation was included as a single variable going from extremely liberal (1) to extremely conservative (7).

Predictors that showed no meaningful relationship with willingness to donate under any of the scenarios – determined by parameter estimates close to null with corresponding *p*-values greater than 0.05 – were dropped from the model. (See Table 1) For consistency in presentation and interpretation, the final model for each scenario included the same set of predictors that showed a meaningful relationship with willingness to donate under at least one scenario. Adjusted odds ratios (AOR) and the 95 % confidence intervals associated with each predictor were obtained based on the model parameter estimates. An AOR greater than 1 indicates a participant characteristic positively associated with willingness to give consent, an AOR less than 1 indicates a characteristic negatively associated with willingness to give consent, while holding other characteristics in the model constant. As noted above, all results including descriptive statistics were weighted to correct for stratified sampling designs, non-coverage and non-response.

## Results

We found an overall shift in willingness to donate using blanket consent when respondents were asked to consider scenarios that raised the possibility of NWIs (Tomlinson et al. 2015). Of the 1,593 participants who responded, a majority – 68 % – agreed with the baseline consent statement: “I would donate tissue samples and medical information to the biobank, so that the biobank can use them for any research study that it allows, without further consent from me.” In all but one of the NWI scenarios – the exception being “stem cells” – the willingness to donate using blanket consent diminished significantly. The percent willing to donate in each (with the *p*-value of the difference from the baseline of 68 %, using conditional logistic regression to examine the paired binary willingness responses for each participant) were: Abortion (49.5 %, *p* < .001), Xenotransplant (64.2 %, *p* = .007), Patents (55.2 %, *P* < .001), Stem cells (70.1 %, *p* = .17), Bioweapons (56.5 %, *p* < .001), Evolution (64.0 %, *p* = .005), Violence gene (58.1 %, *p* < .001). Among the 1,083 respondents who were willing to donate using blanket consent at baseline, 762 (70.4 %) were *unwilling* to donate using blanket consent in at least one NWI scenario (Tomlinson et al. 2015).

### Baseline willingness: willingness to donate using blanket consent

Table 1 reports participant characteristics for all respondents and for those willing and unwilling to donate using blanket consent, *before* they were asked to consider research projects that may raise NWI concerns. Unadjusted comparison between those willing vs. not willing to donate at baseline shows race, ethnicity, education, household income, religion, political views (ranging from extremely liberal to extremely conservative), views on abortion, employment status, ownership of housing, and internet access to be strongly associated with willingness. Attitudes toward biomedical research and residual concern about privacy are also significantly associated with willingness.

In adjusted analyses using logistic models, residual concern about privacy and attitudes toward research were the most powerful predictors: greater concern about privacy was associated with lower willingness to donate (*p* < 0.001, Table 2), while a positive attitude toward research was associated with greater willingness (*p* < 0.001, Table 2). When we controlled for privacy and attitudes toward research, only one of the other variables in our model – race – had an association with willingness to donate: African Americans remained significantly less willing to donate after adjusting for all the other variables, including views on privacy and attitudes towards research (*p* < 0.001).

**Table 2:** Logistic regression predicting willingness to donate using blanket consent (at baseline)^a,b^

	AOR^c^	SE	95 % CI	*p*-value
Age (in years)	1.00	0.00	(0.99, 1.01)	0.57
Female	1.16	0.16	(0.88, 1.52)	0.30
Race				
White	1.00	--	--	--
Black/African American	0.46	0.11	(0.29, 0.73)	< 0.001
Other	1.01	0.28	(0.58, 1.73)	0.98
Hispanic	0.78	0.18	(0.50, 1.22)	0.27
Education^d^	0.93	0.08	(0.79, 1.09)	0.38
Household Income (1–19)	1.01	0.02	(0.98, 1.05)	0.40
Abortion view				
Always legal	1.00	--	--	--
Legal in most circumstances	1.15	0.25	(0.76, 1.76)	0.51
Legal in a few circumstances	1.09	0.23	(0.72, 1.63)	0.69
Always illegal	0.90	0.23	(0.55, 1.47)	0.66
Don’t know	0.96	0.30	(0.52, 1.77)	0.89
Religious affiliation				
Catholic	1.00	--	--	--
Non-Catholic Christian	0.98	0.18	(0.69, 1.41)	0.93
Non-Christian Religions	0.92	0.33	(0.46, 1.84)	0.81
Unaffiliated	1.06	0.24	(0.68, 1.64)	0.80
Do not Know/Refused	0.59	0.23	(0.27, 1.28)	0.18
Political^e^	0.92	0.05	(0.83, 1.02)	0.10
Privacy^f^	0.68	0.04	(0.60, 0.77)	< 0.001
RAQ^g^	1.13	0.01	(1.10, 1.16)	< 0.001

*N* = 1,593

^a^We define blanket consent as a model in which the donor gives permission for unspecified and unknown uses of the specimen at the time of donation. We chose to test a model portraying “blanket consent” with “committee oversight” as a way of focusing on the ethical issue of consenting to future unknown uses of biospecimens – the central issue in the conversation about informed consent for biobanking

^b^Adjusted for post-stratification weights

^c^AOR (Adjusted Odds Ratio) greater than 1 means the participant characteristic is positively associated with willingness to give blanket consent, and less than 1 means the characteristic is negatively associated with willingness to give blanket consent

^d^Range is 1 to 4 (higher is more education)

^e^Range is 1 to 7 (higher is more conservative)

^f^Range is 1 to 5 (higher is more worried)

^g^RAQ is the 11 item Research Attitudes Questionnaire, assessing attitudes toward medical research. Range is 11–66 (a higher score corresponds to more positive attitudes)

### Willingness to donate using blanket consent, when considering scenarios with potential NWI concerns

Table 3 presents the predictors of willingness to donate using blanket consent, after learning the specifics of each of the NWI scenarios. Looking first at the effect of *respondent characteristics* on willingness to donate, we found that across *all* seven scenarios more positive attitudes toward research were associated with significantly greater willingness to donate. Conversely, one’s education and household income were not associated with willingness to donate in *any* of the NWI scenarios. Similarly, one’s religious affiliation had no strong or consistent effect on willingness to donate in the various scenarios.

**Table 3:** Logistic regression predicting willingness to give consent under non-welfare interest scenarios^a^

	Abortion *N* = 1,587	Xeno-transplant *N* = 1,591	Patents *N* = 1,590	Stem cells *N* = 1,590	Bio-weapons *N* = 1,588	Evolution *N* = 1,590	Violence Gene *N* = 1,590
	AOR^b^ (95 % CI)	AOR (95 % CI)	AOR (95 % CI)	AOR (95 % CI)	AOR (95 % CI)	AOR (95 % CI)	AOR (95 % CI)
Age (in years)	0.99* (0.98, 1.00)	1.00 (0.99, 1.00)	1.00 (0.99, 1.00)	0.99 (0.99, 1.00)	0.99* (0.98, 1.00)	0.99* (0.98, 1.00)	0.99* (0.98, 1.00)
Female	0.94 (0.72, 1.23)	0.68* (0.52, 0.88)	0.84 (0.67, 1.07)	0.77 (0.58, 1.01)	0.94 (0.75, 1.20)	0.86 (0.67, 1.10)	0.84 (0.66, 1.06)
Race							
White	1.00 --	1.00 --	1.00 --	1.00 --	1.00 --	1.00 --	1.00 --
Black/African American	0.89 (0.57, 1.40)	0.43** (0.28, 0.67)	1.17 (0.77, 1.77)	0.63 (0.39, 1.02)	1.01 (0.67, 1.52)	0.80 (0.52, 1.23)	0.56* (0.37, 0.85)
Other	1.41 (0.81, 2.47)	0.78 (0.47, 1.30)	0.78 (0.50, 1.24)	1.02 (0.58, 1.79)	1.00 (0.64, 1.57)	0.68 (0.42, 1.09)	0.79 (0.50, 1.24)
Hispanic	0.65 (0.40, 1.03)	0.62* (0.40, 0.97)	0.51* (0.34, 0.77)	0.91 (0.55, 1.49)	0.69 (0.45, 1.06)	0.87 (0.58, 1.33)	0.82 (0.54, 1.24)
Education	0.90 (0.77, 1.06)	0.99 (0.85, 1.16)	0.96 (0.83, 1.10)	0.94 (0.79, 1.11)	0.91 (0.80, 1.05)	0.90 (0.78, 1.04)	0.93 (0.81, 1.07)
Household Income	1.00 (0.96, 1.03)	1.02 (0.99, 1.06)	1.02 (0.99, 1.05)	1.00 (0.97, 1.04)	1.03 (1.00, 1.06)	1.02 (0.99, 1.05)	1.01 (0.98, 1.04)
Abortion view							
Always legal	1.00 --	1.00 --	1.00 --	1.00 --	1.00 --	1.00 --	1.00 --
In most circumstances	0.76 (0.52, 1.11)	0.98 (0.65, 1.47)	1.05 (0.75, 1.49)	0.84 (0.54, 1.32)	1.18 (0.84, 1.67)	1.11 (0.76, 1.63)	0.64* (0.45, 0.91)
In a few circumstances	0.25** (0.17, 0.36)	0.61* (0.41, 0.90)	1.11 (0.79, 1.57)	0.84 (0.55, 1.30)	1.06 (0.75, 1.50)	0.91 (0.63, 1.32)	0.68* (0.48, 0.97)
Always illegal	0.09** (0.05, 0.15)	0.46* (0.29, 0.74)	0.74 (0.48, 1.13)	0.60* (0.36, 0.99)	0.90 (0.59, 1.37)	0.62* (0.39, 0.96)	0.51* (0.33, 0.79)
Don’t know	0.26** (0.15, 0.47)	0.59 (0.33, 1.05)	1.05 (0.61, 1.82)	0.38* (0.21, 0.70)	0.84 (0.47, 1.50)	0.70 (0.40, 1.21)	0.85 (0.49, 1.45)
Religion							
Catholic	1.00 --	1.00 --	1.00 --	1.00 --	1.00 --	1.00 --	1.00 --
Non-Catholic Christian	0.79 (0.57, 1.11)	1.08 (0.76, 1.53)	0.71* (0.51, 0.98)	0.76 (0.51, 1.11)	0.77 (0.56, 1.06)	0.91 (0.66, 1.27)	1.10 (0.81, 1.51)
Non-Christian Religions	0.82 (0.42, 1.60)	0.84 (0.45, 1.59)	0.64 (0.36, 1.13)	0.71 (0.35, 1.46)	0.61 (0.34, 1.09)	1.12 (0.56, 2.21)	0.79 (0.44, 1.39)
Unaffiliated	1.27 (0.85, 1.91)	1.00 (0.66, 1.52)	0.81 (0.56, 1.18)	0.71 (0.45, 1.12)	0.61* (0.42, 0.89)	1.02 (0.68, 1.53)	0.86 (0.59, 1.24)
Do not Know/ Refused	0.84 (0.38, 1.88)	0.68 (0.30, 1.55)	0.64 (0.30, 1.37)	0.51 (0.24, 1.09)	0.71 (0.35, 1.47)	0.77 (0.38, 1.58)	0.66 (0.33, 1.35)
Political^c^	0.75** (0.68, 0.83)	1.02 (0.92, 1.13)	1.01 (0.93, 1.10)	0.86* (0.77, 0.95)	1.02 (0.94, 1.12)	0.86* (0.79, 0.95)	0.96 (0.88, 1.05)
Privacy^d^	0.98 (0.87, 1.11)	0.81** (0.72, 0.91)	0.91 (0.82, 1.02)	0.80** (0.70, 0.90)	0.90 (0.81, 1.00)	0.88* (0.79, 0.99)	1.00 (0.89, 1.11)
RAQ^e^	1.09** (1.07, 1.12)	1.12** (1.09, 1.14)	1.09** (1.07, 1.11)	1.13** (1.10, 1.16)	1.09** (1.07, 1.12)	1.09** (1.07, 1.11)	1.09** (1.07, 1.12)

^a^Adjusted for post-stratification weights

^b^AOR (Adjusted Odds Ratio) greater than 1 means the participant characteristic is positively associated with willingness to give blanket consent, and less than 1 means the characteristic is negatively associated with willingness to give blanket consent

^c^Range is 1 to 7 (higher is more conservative)

^d^Range is 1 to 5 (higher is more worried)

^e^RAQ is the 11 item Research Attitudes Questionnaire, assessing attitudes toward medical research. Range is 11–66 (a higher score corresponds to more positive attitudes)

* *p* < .05

** *p* < .001

Older age reduced willingness to donate in four of the seven NWI scenarios: abortion, bio-weapons, evolution, and violence gene. Residual concern with privacy – strongly associated with lower willingness to donate at baseline (i.e. before exposure to NWIs) – decreased willingness to donate in three of the seven scenarios – xenotransplant, stem cells, and evolution – and was marginally significant (*p* = .06) in the bioweapons scenario. African American identity – another variable strongly associated with unwillingness to donate at baseline – was a significant independent predictor of decreased willingness to donate in *two* NWI scenarios: xenotransplantation and the search for a violence gene.

It is also instructive to look at how, and where, each *scenario* influenced willingness to donate. Two NWI scenarios, patents and bioweapons, diminished willingness to donate by more than 10%age points in the overall sample, but proved to be more or less “non-partisan” in their effect on willingness to donate. That is, respondent characteristics that we would expect to exert influence here – one’s political views and view on abortion – were not associated with decreased willingness to donate, and religion had a minimal effect. On the other hand, the stem cell scenario, which did *not* reduce willingness to donate in the survey, significantly reduced willingness to donate among political conservatives and those with residual concerns about privacy (Table 3).

## Discussion

Our national survey shows that concern with one’s non-welfare interests (NWIs) has a significant effect on willingness to donate to a biobank using blanket consent (Tomlinson et al. 2015). More than 70% of the respondents who were willing to donate using a blanket consent – before being told of the possibility of research that could raise moral, religious, or cultural concerns – became unwilling to donate with a blanket consent in at least one of seven proposed research scenarios associated with NWIs. Thus, when they are made apparent, NWI concerns matter to most people. But not unexpectedly, there is variation in which NWI scenarios matter to which people. Some NWI scenarios – like those involving patents and bioweapons – are of general concern to potential donors, unrelated to a person’s demographic and attitudinal characteristics, while others are of particular concern to certain subgroups.

These data suggest that the current policy of asking potential donors to consent to all future uses of their donations may be empirically and ethically shortsighted. Empirically, we know that NWIs matter. Given our findings and the many future unknown uses of biobanked materials, it is clear that this is an issue that will affect virtually all donors. Ethically, we know that informed consent is problematic if information material to the decision to be made is withheld. Since we know that NWIs are material for most donors, a policy that deliberately avoids any mention of potential donor concerns could undermine the adequacy of the consents given by some donors.

The challenge for biobanks is to design a set of policies that can account for the varied levels of concern about NWIs found in the population. Some may argue that concerns that matter to only a minority of donors are too costly to be addressed at all by a biobank. There are two problems with this conclusion, one specific and one general. In the first instance, policies based solely on the concerns of the majority – determined by surveys or representation by groups in power – will discourage donations from minority groups who may have good reasons to be wary of research, depriving biobanks of donations representing that subgroup. Second, ignoring concerns held by a minority of citizens overlooks the potential positive impact of a transparent public information policy that promotes trust in the work of science.

Our study confirms that trust is critical in the decision to donate (Critchley et al. 2015; Ridgeway et al. 2013). A positive attitude toward biomedical research in general – as measured by the RAQ – was consistently associated with *increased* willingness to donate, both before and after being exposed to scenarios with NWI concerns. Although not as consistent an effect, residual concern with privacy – i.e., trust that researchers will protect personal information – was associated with *decreased* willingness to donate. We also found that African Americans had concerns about donating that remained after controlling for attitudes toward research and concerns with privacy. This latter finding suggests a lack of access to, or unfamiliarity with, research institutions on the part of African Americans (Langford et al. 2014), or distrust in scientific research among members of this group (Corbie-Smith et al. 2002; Braunstein et al. 2008; McDonald et al. 2014), or both.

Given the sheer range and varied distribution of NWI’s among the population, we cannot expect informed consent to do all the ethical work required to promote trust in biobanking. The informed consent process can be improved, but respect for, and accommodation of, donors’ NWI’s need not require longer and more complicated consent forms. The imperative of transparency should extend beyond consent to include policies for disclosure of research being conducted under the auspices of a biobank. Potential donors and the wider public must be given easy access to information about the types of research being done with donations to biobanks. Such transparency can inform decisions about donation in ways that will never be captured in a consent form, and equally important, it can inform donors’ later decisions on whether to withdraw.

Our emphasis on transparency is critical because research ethics regulations are often inadequate. At present in the United States, Institutional Review Board (IRB) oversight of biobank research is limited to determining if it does or does not constitute research on human subjects. And even under the proposed new rules for IRBs in the US, these committees will have no role after the required blanket consent form is approved. To respect and accommodate NWIs we must look beyond regulatory schemes and toward widespread adoption of practices that demonstrate concern for the whole range of donor NWIs, signaling the trustworthiness of research and dispelling worries that diminish the willingness to donate.

Our research has limitations. Although we used a probability-based internet panel to recruit our respondents, the response rate was just over 60%. While this presents a challenge to the external validity of our findings, all analyses were weighted to correct for the stratified sampling designs and other sources of survey errors including non-coverage and non-response. Internal validity may have been compromised by the succinct nature of our descriptions of biobanks and the NWI scenarios. For example, we provided only a brief description of the ethics committee oversight; an actual consent form might include additional details about this oversight that would lessen participants’ concerns. We did pilot test these descriptions and concluded that more detailed descriptions would reduce our response rate and increase the likelihood of varied and unpredictable interpretations on the part of respondents. Also, our selection of NWI scenarios, although based on the literature, was such that, given the heterogeneity of responses to various scenarios, we cannot infer the responses to other potential NWI scenarios. Finally, our respondents were “hypothetical donors,” and we know that willingness to donate reported on a survey does not always correlate with willingness to donate in real life situations [Johnsson et al., 2010]. However, it is not clear that “real” willingness to donate is a more accurate measure of willingness: it may well be that in a clinical or research setting individuals feel additional social pressure to donate or be overwhelmed by lengthy and complex consent forms.

Our research confirms that NWI concerns are real and that they influence one’s willingness to donate to a biobank. Ignoring these concerns is problematic, ethically and pragmatically. It is ethically problematic to gain consent while withholding information that matters to those giving their consent, and pragmatically, it seems shortsighted to use a consent process and public information policy that could undermine public trust in research. Is it possible to find a way to take these interests into account without incurring prohibitive costs? And is it possible to both alert people to research they might find concerning, and at the same time assure them of the positive contributions made possible by their participation? We believe such a goal is achievable but in order to improve the consent processes used by, and the transparency of, biobanks it is necessary to consult the public about their attitudes toward NWIs and their views about whether and how these should be accommodated by biobanks.

## Abbreviations

NWI: Non-welfare interest
RAQ: Research attitudes questionnaire
AOR: Adjusted odds ratio
